# An increased McKibbin Index is associated with anterosuperior labral hypertrophy in the context of non‐dysplastic hips

**DOI:** 10.1002/jeo2.70748

**Published:** 2026-05-20

**Authors:** Octavian Andronic, Thaddaeus Muri, Felix Öttl, Dimitris Dimitriou, Armando Hoch, Patrick O. Zingg

**Affiliations:** ^1^ Department of Orthopedics Balgrist University Hospital Zurich Switzerland

**Keywords:** femoral anteversion, hip preservation, labral hypertrophy, McKibbin Index

## Abstract

**Purpose:**

This study aims to investigate the relationship between anterosuperior labral hypertrophy and other morphological parameters in hips with radiographically sufficient lateral coverage. Our hypothesis is that anterior undercoverage or combined high values of femoral torsion and acetabulaer version, represented as an increased McKibbin Index, would be associated with localized anterior labral hypertrophy.

**Methods:**

This was a retrospective case‐control study at a single institution. A consecutive cohort was screened between January 2014 and September 2024 that received either magnetic resonance imaging (MRI) or MR‐arthrography of the hip. Only hips with normal lateral centre edge angle values (between 25° and 40°) were included. Previously described radiographic parameters were evaluated: femoral torsion, acetabular version, Tönnis angle, anterior wall index (AWI), posterior wall index (PWI), McKibbin Index and labral height‐to‐length ratio on axial planes as a measure of anterior labral hypertrophy. Statistical analysis included *χ* ^2^ test, Fisher's exact test, Pearson and partial Pearson correlation analysis, as well as univariate and multiple regression analysis. A total of 132 patients were included in the study, of whom 51 (39%) exhibited anterosuperior labral hypertrophy (labral height‐to‐length ratio of less than 1:2).

**Results:**

The McKibbin Index demonstrated a significant negative correlation with the labral height‐to‐length ratio on the axial plane (*r* = –0.182, *p* = 0.018), indicating greater labral hypertrophy with increased combined femoral and acetabular anteversion. Femoral torsion showed a similar association (*r* = –0.167, *p* = 0.028). Using the multiple regression analysis, the McKibbin Index remained significant in variable exclusion testing (*p* = 0.037), supporting its relevance to labral morphology.

**Conclusion:**

Our findings highlight the correlation of the increased McKibbin index with anterosuperior labral hypertrophy and suggest relevant implications of the global rotational alignment, including both the acetabular version and femoral torsion on joint and labral biomechanics.

**Level of Evidence:**

Level IV.

**Abbreviations**
3D: three‐dimensional
AC Index: acetabular index
AWI: anterior wall index
BMI: body mass index
COS: crossover sign
DDH: developmental dysplasia of the hip
FAI: femoroacetabular impingement
ISS: ischial spine sign
LCEA: lateral centre edge angle
MRI: magnetic resonance imaging
PWI: posterior wall index
PWS: posterior wall sign

## INTRODUCTION

The hip labrum has emerged as a sensitive marker of biomechanical stress in various forms of femoroacetabular impingement (FAI) and developmental dysplasia of the hip (DDH) [4, 13, 31]. While labral hypertrophy is commonly observed in dysplastic hips with lateral acetabular undercoverage, less is known about its occurrence and importance in patients who demonstrate sufficient lateral coverage.

Recent work has highlighted the limitations of using lateral centre edge angle (LCEA) as the sole radiographic indicator of acetabular sufficiency [4, 16, 20]. While anterior wall coverage, characterized by the anterior wall index (AWI), is a more nuanced measure of anterior joint morphology [27, 32], it does not account for abnormal femoral torsion [25]. The acetabular labrum often undergoes adaptive changes in response to altered contact mechanics; hypertrophy, in particular, may represent a compensatory response to subtle anterior undercoverage. Recent contributions by Andronic et al. [2, 3] have emphasized the clinical and radiographic variability within borderline dysplasia and the biomechanical importance of regional acetabular geometry. In addition, a study by Karisch et al showed worse PROM's in borderline dysplasia compared to true dysplasia, underscoring the clinical relevance for a better understanding of this specific population [15].

Moreover, rotational alignment, particularly as described by the femoral torsion angle and acetabular version, has been implicated in labral pathology. The McKibbin Index, a summation of femoral and acetabular version, has been associated with both hip instability and FAI in recent studies [19]. However, its relevance to soft tissue adaptation, such as labral hypertrophy, remains unclear. While some studies have suggested an association between excessive femoral anteversion and labral hypertrophy or tears [18, 33] others have failed to replicate these findings when regional acetabular parameters are considered [9].

This study aims to investigate the relationship between anterosuperior labral hypertrophy and other morphological parameters in hips with radiographically sufficient lateral coverage. By isolating hips with LCEA values between 25° and 40°, we sought to assess whether any acetabular or femoral morphological parameters associate with anterosuperior labral hypertrophy in non‐dysplastic hips. We hypothesize that either a decreased AWI, an increased femoral antetorsion, an increased acetabular anteversion or their combination represented as an increased McKibbin index would be associated with localized anterior labral hypertrophy as marker for anterior stress due to anterior instability on the hip joint. Highlighting these associations may facilitate identification of hips with anterior instability and lead to a better understanding of the influence of the rotational hip alignment on the anterior labrum and thereby helping to close that gap in the literature. Establishing these associations may lead in the long run to a pronounced focus on those parameters in patients with anterior hip pain in cases of normal lateral coverage and may influence further studies concerning the surgical treatment strategy in those cases.

## METHODS

This retrospective case‐control study was performed at a single institution and conducted in accordance with the STROBE Protocol [36] as well as Swiss and international law requirements (Good Clinical Practice, according to the European Medicines Agency). Ethical board's approval was obtained from the Ethical Committee of the Canton of Zurich, Switzerland (ID: BASEC Nr. 2025‐00248). Data collection was performed between February 2025 and June 2025.

Data were collected from a cohort of patients between January 2014 and September 2024 who received either a 3.0 T magnetic resonance imaging (MRI) or MR‐arthrography of the hip at our institution. The time interval was chosen following the standardization of MRI protocols, with fixed lower limb positioning to allow femoral torsion quantification [13, 31].

All radiology reports were screened for ‘femoral torsion’ by utilizing a locally run large language model (LLAMA 3.2, 3B, Meta) [38] Utilizing this method, we achieved during a manual check of the data an accuracy of 91% from the LLM based on a randomly selected subset of 100 patients. All hips selected by the LLM were eventually included following manually chart review and according to the inclusion/exclusion criteria.

All hips from patients between 18 and 40 years and with radiographically closed growth plates were considered eligible. Hips with complete imaging were further selected, meaning a correctly performed anteroposterior pelvic radiograph [32], MRI or MR‐arthrography [28, 35, 39] with axial slices of the hip and pelvis allowing the calculation of the femoral torsion according to Sutter et al. [31] and also available pelvic axial images to allow the calculation of the acetabular version according to Hetsroni et al. [13]. Only hips with normal LCEA values, between 25° and 40°, were selected [23, 34].

All hips with deformities resulting from genetic abnormalities, tumours, trauma, slipped capital femoral epiphysis (SCFE), femoral head osteonecrosis [5], previous surgeries, Perthes disease or a Tönnis grading >1 [17] were excluded.

### Radiographic evaluation

**Figure 1:**
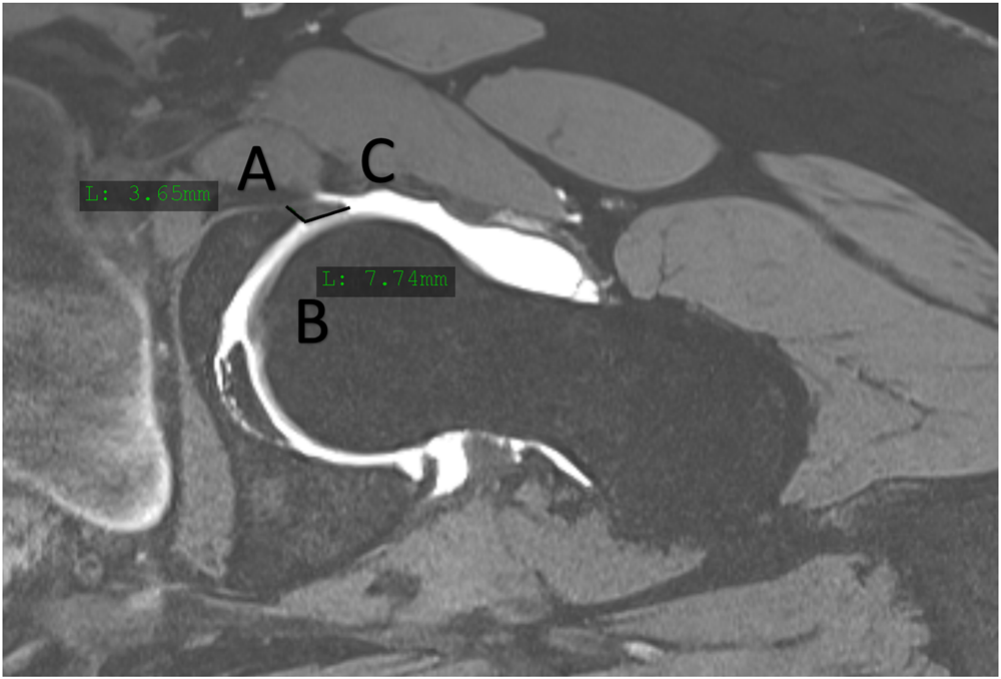
Examples of anterosuperior labral hypertrophy (height [AB] to length [AC] ratio ≤1:2) measured on axial MRI imaging.

As absolute labral size may vary based on individual size, labral hypertrophy was defined based on a previously described ratio measured on the MRI scan [33]. It was considered hypertrophic when length was twice as large as the height (height‐to‐length ratio 1:2) (Figure 1). Labrum assessment on MR is a proven method with good intra‐ and interobserver reliability [1, 30]. Labral lesions were classified according to Villar et al. [18]. For description of labral location, the 12 o'clock system was used: 12 o'clock position being superior, 3 o'clock anterior, 6 o'clock inferior (transverse ligament) and 9 o'clock posterior. For easier analysis and presentation, the data of the left hips were converted into right hips and presented in a clockwise fashion.

**Figure 2:**
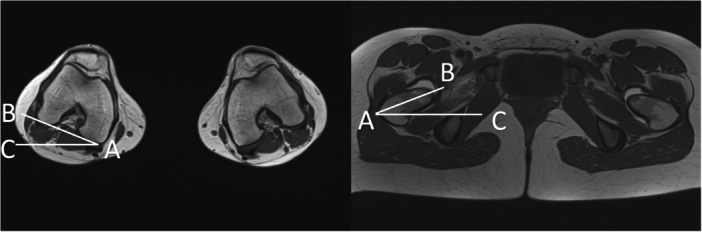
Measurement of the femoral torsion between the centre of the base of the femoral neck at its narrowest point and the condylar axis (AC—horizontal line, AB—condylar axis, left image; AB—femoral neck axis, right image).

Femoral version was measured on the MRI between the centre of the base of the femoral neck at its narrowest point and the condylar axis, as previously described [31] (Figure 2). Definitions and categorization for femoral version varied in the literature. Previous thresholds for decreased femoral version were <10° [7, 34], <5° [8, 11] and <0° [16] and for increased femoral version were >22° [7] and >25° [8, 9, 34]. For the purpose of this study, a threshold value of >22° was considered as an increased femoral torsion, whereas values between 2° and 22° were considered normal [31].

**Figure 3:**
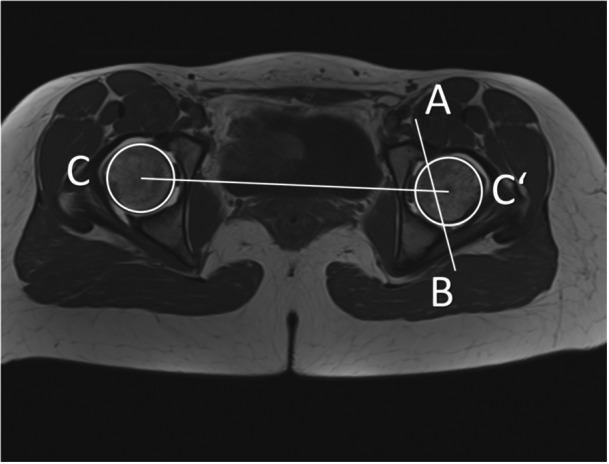
Measurement of the acetabular version is defined as the angle between a sagittal line perpendicular to a line connecting the centres of the femoral head (CC′) and a line connecting the anterior and posterior acetabular rim on the level of the femoral head centre (AB).

Apart from the assessment of classical signs of acetabular retroversion on the pelvic anteroposterior conventional radiograph (posterior wall sign [PWS], crossover sign [COS] and ischial spine sign [ISS] [3, 32]), the central acetabular version was measured on the MRI according to the method described by Hetsroni et al. [13] who defined the central acetabular version as the angle between a sagittal line and a line connecting the anterior and posterior acetabular rim on the level of the femoral head centre (Figure 3). Pelvic positioning was neutralized, connecting the bilateral centre of the femoral head on axial images as described previously [20]. Normal central acetabular version was defined in the range between 10° and 25° [19]. The McKibbin index was defined as the sum of femoral torsion and acetabular version. An increased McKibbin index was defined as >50° and a decreased McKibbin index as <20° [19].

Anterior and posterior hip coverage was also assessed using the acetabular wall index (anterior—AWI; posterior—PWI) on anteroposterior radiographs, as described by Siebenrock et al. [27]. Previous studies showed good intra‐ and interobserver reliabilities for the mentioned measurements, therefore no repeat analysis for reliability was performed as part of the present study. For AWI and PWI intra‐ and interobserver reliabilities were shown to be 0.94 and 0.99, respectively, 0.81 and 0.97 for the PWI in the literature [27].

Schmaranzer et al showed an intraobserver reproducibility of 0.962 and an interobserver reliability of 0.939 for femoral version [26], while Meier et al. presented an intraobserver reproducibility of 0.8 and interobserver agreement of 0.75 for central acetabular version [21].

All radiological measurements have been performed by two independent orthopaedic residents trained in musculoskeletal imaging. In cases of uncertainty, consensus was reached after consultation with a senior orthopaedic consultant.

### Statistical analysis

Data extraction variables included demographics (age, gender, side, body mass index [BMI]) and radiographic parameters (LCEA, acetabular index [AC index], AWI, PWI, femoral torsion, acetabular retroversion [as combined: COS, ISS and PWS], labral height and width, presence and location of labral lesions).

For the calculation of differences between continuous variables (absolute numerical values such as femoral torsion or acetabular version), a two‐tailed matched‐pairs *t* test was performed to account for no directional hypothesis. For the calculation of differences in binary/categorical outcomes, a *χ* ^2^ test was performed. Pearson's correlation coefficient was used to interpret the strength of correlation according to previously published papers (e.g., small: < 0.2, medium: 0.2–0.5, large > 0.8) [29]. A priori power analysis for the fixed‐model linear regression analysis (total 6 predictors stepwise: LCEA, AC Index, age and AWI, femoral torsion and McKibbin Index as a continuous variable) showed a required total sample size of 119 hips with a medium effect size of 0.15 (Cohen), Power 95% and alpha error probability 5%. A *p* value of <0.05 was considered significant. Statistical analysis was performed using the G Power software (3.1) [10].

## RESULTS

A total of 132 patients were finally included in the study (Table 1), of whom 51 (38.6%) exhibited labral hypertrophy, defined as a labral height‐to‐length ratio of less than 1:2. Among patients with labral hypertrophy, 64% were female, compared to 52% in the non‐hypertrophy group (Table 2). The average age of the study population was 26.5 years.

**Table 1:** Demographics and baseline characteristics.

	Average/Total values	Standard deviation
Age	26.5 years	±6.1 years
Gender	57 males 85 females	NA
LCEA°	29.8°	±3.8°
AC Index°	3.5°	±2.4°

Abbreviations: AC Index, acetabular index (Tönnis angle); LCEA, lateral centre edge angle of Wiberg; NA, not applicable.

**Table 2:** Radiographic findings.

	Average	Standard deviation	Observations
Femoral torsion	24.0°	±8.9°	*N* = 67 with FT over 25°
Acetabular version	16.4°	±6.5°	
McKibbin Index	40.4°	±11.9°	*N* = 26 with McKibbin Index over 50°
AWI	0.52	±0.16	
PWI	0.93	±0.19	
Labral height‐to‐length ratio (axial MRI)	0.57	±0.13	*N* = 51 with values less than 0.5 (labral hypertrophy)

Abbreviations: AV, acetabular version; AWI, anterior wall index; FT, femoral torsion; PWI, posterior wall index.

McKibbin Index showed a statistically significant negative correlation with the labral height‐to‐length ratio (*r* = –0.18, *p* = 0.018) in the Pearson correlation analysis, indicating that higher combined femoral and acetabular anteversion is associated with increased labral hypertrophy (Table 3). Femoral torsion alone also demonstrated a significant negative correlation with the labral height‐to‐length ratio (*r* = –0.17, *p* = 0.028).

**Table 3:** Correlation of morphologic factors with labral height‐to‐length ratio (bivariate Pearson analysis).

	*r* value	*p* value
Age at MRI	−0.14	*0.054*
LCEA	0.04	0.334
AC Index	−0.12	*0.082*
Acetabular retroversion	0.04	0.333
Femoral torsion	−0.17	**0.028**
McKibbin Index	−0.18	**0.018**
AWI	0.09	0.159
PWI	0.05	0.283

*Note*: Bold values indicate statistically significant.

Abbreviations: AC Index, acetabular index (Tönnis angle); AWI, anterior wall index; LCEA, lateral centre edge angle of Wiberg; MRI, magnetic resonance imaging; PWI, posterior wall index.

Age at MRI (*r* = −0.14, *p* = 0.054) and the AC Index (*r* = −0.12, *p* = 0.082) did not reach statistical significance but showed negative associations with the labral height‐to‐length ratio (Table 3).

To identify independent predictors of labral hypertrophy, a linear multiple regression analysis was performed to evaluate for predictors of labral height‐to‐length ratio as a quantitative measure of labral hypertrophy. In the final model including AWI, AC Index, LCEA, age, femoral torsion and McKibbin Index, the McKibbin Index remained directionally consistent in the regression analysis (*β* = –0.137) (Table 4). Importantly, in the variable exclusion statistics, the McKibbin Index still emerged as a significant contributor (*β* = −0.187, *p* = 0.037).

**Table 4:** Correlation of morphologic factors with labral height‐to‐length ratio (linear multiple regression analysis).

	*r* value	*p* value
Age at MRI	−0.113	0.197
LCEA	−0.094	0.391
AC Index	−0.158	0.122
Femoral Torsion	−0.050	0.760
McKibbin Index	−0.137	0.433
AWI	0.017	0.869

Abbreviations: AWI, anterior wall index; AC Index, acetabular index (Tönnis angle); LCEA, lateral centre edge angle of Wiberg; MRI, magnetic resonance imaging.

## DISCUSSION

A significant association between anterosuperior labral hypertrophy and an increased McKibbin index in our cohort was found. Additionally, in the multivariate model, the McKibbin index was the sole independent predictive value for labral hypertrophy, as measured quantitatively by the labral height‐to‐length ratio on the axial MRI, suggesting its potential relevance even when considering other confounders. Therefore, the hypothesis was partially confirmed regarding the McKibbin Index but was not supported regarding the AWI or the femoral torsion, respectively, the acetabular version. Our finding suggests combined increased femoral torsion and acetabular version are associated with labral hypertrophy. Clinically thinking, it may be a consequence of labral adaptation due to labral overload, yet the biomechanical studies are missing to support this theory. Additionally, supporting this association, femoral torsion alone was near‐significantly associated with anterior labral hypertrophy, a finding that could achieve a significant result in a larger sample size.

Even though our findings showed no statistical significance for age and AC Index, we would not like to rule out completely an association between an increased age or a more inclined acetabulum with labral adaptations, especially age might have achieved a statistical significance in larger study groups, regarding the *p* value of 0.054.

Our results depict a new association of rotational malalignment with anterior labral hypertrophy, expanding further the previous observation of lateral labral hypertrophy in hip dysplasia [33].

Prior studies by Boschung et al. [6] and Lerch et al. [19] have shown that McKibbin extremes are associated with posterior or anterior extra‐articular impingement, which may have implications for labral stress and morphology. However, no implications of rotational malalignment on patients with localized anterior instability were described. Previous work has shown that lateral labral hypertrophy may represent an adaptive response to subclinical instability [19, 20, 27] as seen in dysplastic hips.

Therefore, future clinical studies may focus on the benefit of adjunct correction osteotomy in symptomatic hips with an increased McKibbin index and the corresponding radiological finding of anterior labrum hypertrophy as observed in our cohort, as the joint load distribution and anterior instability would remain unaddressed in a scenario of isolated labral management, as well as mid‐ to long‐term follow‐up studies similar to the joint arthroplasty research are needed to compare the outcome according the surgical strategy [14]. Notably, regarding the fact that arthroscopic hip surgery seems to be a viable option compared to periacetabular osteotomy in specific cases of borderline dysplasia [24]. In addition, newer clinical assessments, such as the lateral hip instability test [37], may assist in clinical decision‐making and further streamline patient‐specific surgical planning.

Regarding possible studies in the future assessing the influence of an adjunct femoral osteotomy, the critical biomechanical effect of the alignment of the trochanter major on gait kinematics must be taken into account [25, 40]. Recent simulation studies using three‐dimensional (3D) models demonstrated that subtrochanteric corrective osteotomies shift the trochanter major anteriorly or posteriorly by approximately one centimetre per 10° of torsional correction [12]. This shift modifies the abductor lever arm and subtly alters the abduction moment, which may contribute to impaired muscle efficiency and compensatory gait changes [25, 40].

This study has several limitations. First, acetabular morphology was assessed using two‐dimensional static imaging, which may not fully capture the 3D complexity of acetabular anatomy. Using newer technology, such as deep learning 3D MRI [22] model, should be considered for future studies.

Further, the lack of dynamic assessment limits our understanding of how static morphology relates to in vivo joint mechanics. As there was no follow‐up imaging, we could not evaluate changes over time based on imaging, but our results included age as a confounder, and findings were adjusted for age as well. Despite these limitations, the study's strengths include the use of a consecutive patient cohort, strict exclusion criteria to ensure a morphologically homogeneous population and robust statistical methodology with both multivariate and Pearson correlation analyses.

In conclusion, our findings highlight the correlation of a high McKibbin index with anterosuperior labral hypertrophy in hips with normal lateral coverage. It underlines the importance of the global rotational alignment, including both the acetabular version and femoral torsion, for understanding the hip joint and labral morphology.

## AUTHOR CONTRIBUTIONS

Octavian Andronic and Patrick O. Zingg designed the research methodology and the research question. Armando Hoch managed the research team and assisted in the data acquisition. Felix Öttl and Thaddaeus Muri performed data collection, ethics application and data analysis with statistical analysis, as well as drafted the first manuscript. All authors critically appraised the scientific approach and the final draft of the manuscript. All authors read, edited and approved the final manuscript.

## CONFLICT OF INTEREST STATEMENT

The authors declare no conflicts of interest.

## ETHICS STATEMENT

The presented study was conducted in accordance with Swiss and international law requirements. Ethical board's approval was obtained from the Ethical Committee of the Canton of Zurich, Switzerland (ID: BASEC Nr. 2025–00248).

## Supporting information

Flow Chart femoral Torsion.

STROBE Checklist.

## Data Availability

The data that support the findings of this study are available from the corresponding author upon reasonable request.
