# Supplementary figures and images for: An increased McKibbin Index is associated with anterosuperior labral hypertrophy in the context of non‐dysplastic hips
Source: J Exp Orthop. 2026 May 20;13(2):e70748. doi: 10.1002/jeo2.70748 (PMC13239846; doi:10.1002/jeo2.70748)

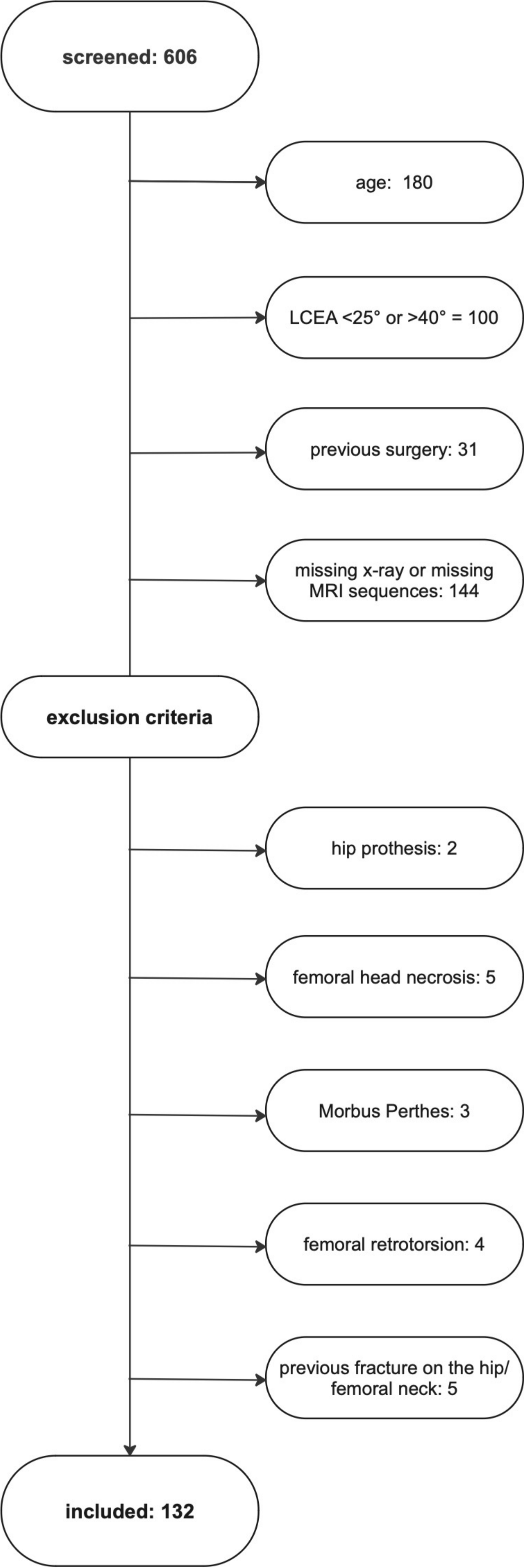

Supplement: Supplementary file 1 — Flow Chart femoral Torsion. [file JEO2-13-e70748-s001.pdf]
