# Supplementary material for: An increased McKibbin Index is associated with anterosuperior labral hypertrophy in the context of non‐dysplastic hips
Source: J Exp Orthop. 2026 May 20;13(2):e70748. doi: 10.1002/jeo2.70748 (PMC13239846; doi:10.1002/jeo2.70748)
Supplement: Supplementary file 2 — STROBE Checklist. [file JEO2-13-e70748-s002.docx]

STROBE Statement—checklist of items that should be included in reports of observational studies

|  | Item No. | Recommendation | Page  No. | Relevant text from manuscript |
| --- | --- | --- | --- | --- |
| **Title and abstract** | 1 | (*a*) Indicate the study’s design with a commonly used term in the title or the abstract | 1 |  |
|  |  | (*b*) Provide in the abstract an informative and balanced summary of what was done and what was found | 2 | This study aims to investigate the relationship between anterosuperior labral hypertrophy and other morphological parameters in hips with radiographically sufficient lateral coverage. Our hypothesis is that anterior undercoverage or combined high values of femoral torsion and acetabulaer version, represented as an increased McKibbin index, would be associated with localized anterior labral hypertrophy. This was a retrospective case-control study at a single institution |
| Introduction | | | |  |
| Background/rationale | 2 | Explain the scientific background and rationale for the investigation being reported | 4 | The hip labrum has emerged as a sensitive marker of biomechanical stress in various forms of femoroacetabular impingement (FAI) and developmental dysplasia of the hip (DDH).While labral hypertrophy is commonly observed in dysplastic hips with lateral acetabular undercoverage, less is known about its occurrence and importance in patients who demonstrate sufficient lateral coverage.Recent work has highlighted the limitations of using lateral center-edge angle (LCEA) as the sole radiographic indicator of acetabular sufficiency. While anterior wall coverage, characterized by the anterior wall index (AWI), is a more nuanced measure of anterior joint morphology, it does not account for abnormal femoral torsion. The acetabular labrum often undergoes adaptive changes in response to altered contact mechanics; hypertrophy, in particular, may represent a compensatory response to subtle anterior undercoverage. Recent contributions by Andronic et al.,have emphasized the clinical and radiographic variability within borderline dysplasia and the biomechanical importance of regional acetabular geometry.  Moreover, rotational alignment, particularly as described by the femoral torsion angle and acetabular version, has been implicated in labral pathology. The McKibbin Index, a summation of femoral and acetabular version, has been associated with both hip instability and femoroacetabular impingement in recent studies. However, its relevance to soft tissue adaptation, such as labral hypertrophy, remains unclear. |
| Objectives | 3 | State specific objectives, including any prespecified hypotheses | 5 | We hypothesize that either a decreased AWI, an increased femoral antetorsion, an increased acetabular anteversion or their combination represented as an increased McKibbin index would be associated with localized anterior labral hypertrophy. Highlighting these associations may facilitate identification of hips with anterior instability and guide surgical treatment. |
| Methods | | | |  |
| Study design | 4 | Present key elements of study design early in the paper |  |  |
| Setting | 5 | Describe the setting, locations, and relevant dates, including periods of recruitment, exposure, follow-up, and data collection | 6 | Data was collected from a consecutive cohort of patients between 01/2014 and 09/2024 that received either a 3.0T magnetic resonance imaging (MRI) or MR-arthrography of the hip at our institution. Patient identification was performed by utilizing a locally run large language model (LLAMA 3.2, 3B, Meta, California, United States)for initial extraction of the femoral torsion from the radiology report. The responses given by the large language model had then undergone further extraction to isolate the keyword “femoral torsion” at specified values. |
| Participants | 6 | (*a*) *Cohort study*—Give the eligibility criteria, and the sources and methods of selection of participants. Describe methods of follow-up  *Case-control study*—Give the eligibility criteria, and the sources and methods of case ascertainment and control selection. Give the rationale for the choice of cases and controls  *Cross-sectional study*—Give the eligibility criteria, and the sources and methods of selection of participants | 6 | All hips from patients between 18 and 40 years and with radiographically closed growth plates were considered eligible. Hips with complete imaging were further selected, meaning a correctly performed anteroposterior pelvic radiograph, MRI or MR-arthrography with axial slices of the hip and pelvis allowing the calculation of the femoral torsion according to Sutter et al. and also available pelvic axial images to allow the calculation of the acetabular version according to Hetsroni et al. Only hips with normal LCEA values, between 25° and 40°, were selected.  All hips with deformities resulting from genetic abnormalities, tumors, trauma, slipped capital femoral epiphysis (SCFE), femoral head osteonecrosis previous surgeries, Perthes disease or a Tönnis grading >1 were excluded.  A total of 132 patients were finally included in the study (**Table 1**), of whom 51 (38.6%) exhibited labral hypertrophy, defined as a labral height-to-length ratio of less than 1:2. Among patients with labral hypertrophy, 64% were female, compared to 52% in the non-hypertrophy group (**Table 2**). The average age of the study population was 26.5 years. |
|  |  | (*b*) *Cohort study*—For matched studies, give matching criteria and number of exposed and unexposed  *Case-control study*—For matched studies, give matching criteria and the number of controls per case |  |  |
| Variables | 7 | Clearly define all outcomes, exposures, predictors, potential confounders, and effect modifiers. Give diagnostic criteria, if applicable | 7 | Measurement of femoral version, AC Index, LCEA, acetabular version, McKibbin Index, Labral hypertrophy, AWI, PWI, age, |
| Data sources/ measurement | 8* | For each variable of interest, give sources of data and details of methods of assessment (measurement). Describe comparability of assessment methods if there is more than one group | 7/8 | Labral hypertrophy was defined based Toeft et al 2015, it was considered hypertrophic when length was twice as large as the height (height-to-length ratio 1:2. Labral lesions were classified according to Villar et al.  Femoral version was measured between the center of the base of the femoral neck at its narrowest point and the condylar axis, as previously described by Sutter et al. For this study, a threshold value of >22° was considered as an increased femoral torsion whereas values between 2° to 22° were considered normal.  Apart from the assessment of classical signs of acetabular retroversion on the pelvic anteroposterior conventional radiograph (posterior wall sign (PWS), crossover sign (COS) and ischial spine sign (ISS), the central acetabular version was measured according to the method described by Hetsroni et al. who defined the central acetabular version as the angle between a sagittal line and a line connecting the anterior and posterior acetabular rim on the level of the femoral head center (Fig. 2). Normal central acetabular version was defined in the range between 10° and 25° as described by Lerch et al. The McKibbin index was defined as the sum of femoral torsion and acetabular version. An increased McKibbin index was defined as >50° and a decreased McKibbin index as <20°.  Anterior and posterior hip coverage was also assessed using the acetabular wall index (anterior - AWI; posterior - PWI) on ap radiographs, as described by Siebenrock et al. |
| Bias | 9 | Describe any efforts to address potential sources of bias |  | Strict inclusion/exclusion criteria. Standardized radiology imaging after 01/2014. Manually chart review before the inclusion/exclusion. |
| Study size | 10 | Explain how the study size was arrived at | 8 | A priori power Analysis with the G Power Software 3.1. showed a required total sample size of 119 hips with a medium effect size of 0.15 (Cohen), Power 95% and alpha error probability 5%. A p value of <0.05 was considered significant. |

Continued on next page

| Quantitative variables | 11 | Explain how quantitative variables were handled in the analyses. If applicable, describe which groupings were chosen and why | 8 | For the calculation of differences between continuous variables (absolute numerical values such as femoral torsion or acetabular version), a two-tailed matched paired t-test was performed to account for no directional hypothesis. For the calculation of differences in binary/categorical outcomes a chi-square test was performed. |
| --- | --- | --- | --- | --- |
| Statistical methods | 12 | (*a*) Describe all statistical methods, including those used to control for confounding | 8/9 | Two-tailed matched paired t-test and a chi-square Test was performed. To identify independent predictors a linear multiple regression model was performed. Finally, an variable exclusion model was run as well. The tables with the results are in the text. |
|  |  | (*b*) Describe any methods used to examine subgroups and interactions |  |  |
|  |  | (*c*) Explain how missing data were addressed |  | If patients had missing data (e.g. missing imaging), they were excluded. |
|  |  | (*d*) *Cohort study*—If applicable, explain how loss to follow-up was addressed  *Case-control study*—If applicable, explain how matching of cases and controls was addressed  *Cross-sectional study*—If applicable, describe analytical methods taking account of sampling strategy |  | There was no matching |
|  |  | (*e*) Describe any sensitivity analyses |  | It was not performed |
| Results | | | | |
| Participants | 13* | (a) Report numbers of individuals at each stage of study—eg numbers potentially eligible, examined for eligibility, confirmed eligible, included in the study, completing follow-up, and analysed |  | 660 Screend  Excluded due to:  Age =180  LCEA <25 or >40° =100  Previous surgery = 31  Missing Imaging = 144  Hip prothesis = 2  Femoral head necrosis = 5  M. Perthes = 3  Femoral retrotorsion = 4  Previous fracture /trauma = 5  Included 132 |
|  |  | (b) Give reasons for non-participation at each stage |  | See above |
|  |  | (c) Consider use of a flow diagram |  | See above |
| Descriptive data | 14* | (a) Give characteristics of study participants (eg demographic, clinical, social) and information on exposures and potential confounders | 6 | All between 18-40 years, Age and Gender are presented. |
|  |  | (b) Indicate number of participants with missing data for each variable of interest |  | -- |
|  |  | (c) *Cohort study*—Summarise follow-up time (eg, average and total amount) |  |  |
| Outcome data | 15* | *Cohort study*—Report numbers of outcome events or summary measures over time |  |  |
|  |  | *Case-control study—*Report numbers in each exposure category, or summary measures of exposure | *7* | A total of 132 patients were finally included in the study of whom 51 (38.6%) exhibited labral hypertrophy.. Among patients with labral hypertrophy, 64% were female, compared to 52% in the non-hypertrophy group. The average age of the study population was 26.5 years. |
|  |  | *Cross-sectional study—*Report numbers of outcome events or summary measures |  |  |
| Main results | 16 | (*a*) Give unadjusted estimates and, if applicable, confounder-adjusted estimates and their precision (eg, 95% confidence interval). Make clear which confounders were adjusted for and why they were included |  |  |
|  |  | (*b*) Report category boundaries when continuous variables were categorized |  | There were no categories made. |
|  |  | (*c*) If relevant, consider translating estimates of relative risk into absolute risk for a meaningful time period |  |  |

Continued on next page

| Other analyses | 17 | Report other analyses done—eg analyses of subgroups and interactions, and sensitivity analyses | 9 | To identify independent predictors of labral hypertrophy, a linear multiple regression analysis was performed to evaluate for predictors of labral height-to-length ratio as a quantitative measure of labral hypertrophy. In the final model including AWI, AC Index, LCEA, age, femoral torsion, and McKibbin Index, the McKibbin Index remained directionally consistent in the regression analysis (β = –0.137), (Table 4). Importantly, in the variable exclusion statistics, the McKibbin Index still emerged as a significant contributor (β = -0.187, p = 0.037). |
| --- | --- | --- | --- | --- |
| Discussion | | | | |
| Key results | 18 | Summarise key results with reference to study objectives | 11 | Significant associatin between labral hypertrophy and an increased McKibbin Index in our cohort. McKibbin Index as sole independent predictive value for labral hypertrophy as measured quantitively by labral height-to-length ratio. Trends showing significance for Age and AC Index and labral hypertrophy. |
| Limitations | 19 | Discuss limitations of the study, taking into account sources of potential bias or imprecision. Discuss both direction and magnitude of any potential bias | 11 | This study has several limitations. First, acetabular morphology was assessed using 2D static imaging, which may not fully capture the 3D complexity of acetabular anatomy. Using newer technology, such as deep learning 3-D MRI38 model should be considered for future studies.  Further, the lack of dynamic assessment limits our understanding of how static morphology relates to in-vivo joint mechanics. As there was no follow-up imaging, we could not evaluate changes over time based on imaging, but our results included age as a confounder and findings were adjusted for age as well. Despite these limitations, the study’s strengths include the use of a consecutive patient cohort, strict exclusion criteria to ensure a morphologically homogeneous population, and robust statistical methodology with both multivariate and Pearson correlation analyses. |
| Interpretation | 20 | Give a cautious overall interpretation of results considering objectives, limitations, multiplicity of analyses, results from similar studies, and other relevant evidence | 10 | Therefore, in symptomatic hips with an increased McKibbin index and the corresponding radiological finding of anterior labrum hypertrophy as observed in our cohort, an adjunct correction osteotomy should be considered, as the joint load distribution and anterior instability would remain unaddressed in a scenario of isolated labral management. |
| Generalisability | 21 | Discuss the generalisability (external validity) of the study results | 11 | In conclusion, our findings highlight the correlation of a high McKibbin Index with antero-superior labral hypertrophy and suggests the relevant implications of the global rotational alignment, including both the acetabular version and femoral torsion on joint and labral biomechanics. |
| Other information | |  | | |
| Funding | 22 | Give the source of funding and the role of the funders for the present study and, if applicable, for the original study on which the present article is based | Title page | NO funding was received for the performance of this study. |

*Give information separately for cases and controls in case-control studies and, if applicable, for exposed and unexposed groups in cohort and cross-sectional studies.

**Note:** An Explanation and Elaboration article discusses each checklist item and gives methodological background and published examples of transparent reporting. The STROBE checklist is best used in conjunction with this article (freely available on the Web sites of PLoS Medicine at http://www.plosmedicine.org/, Annals of Internal Medicine at http://www.annals.org/, and Epidemiology at http://www.epidem.com/). Information on the STROBE Initiative is available at www.strobe-statement.org.
